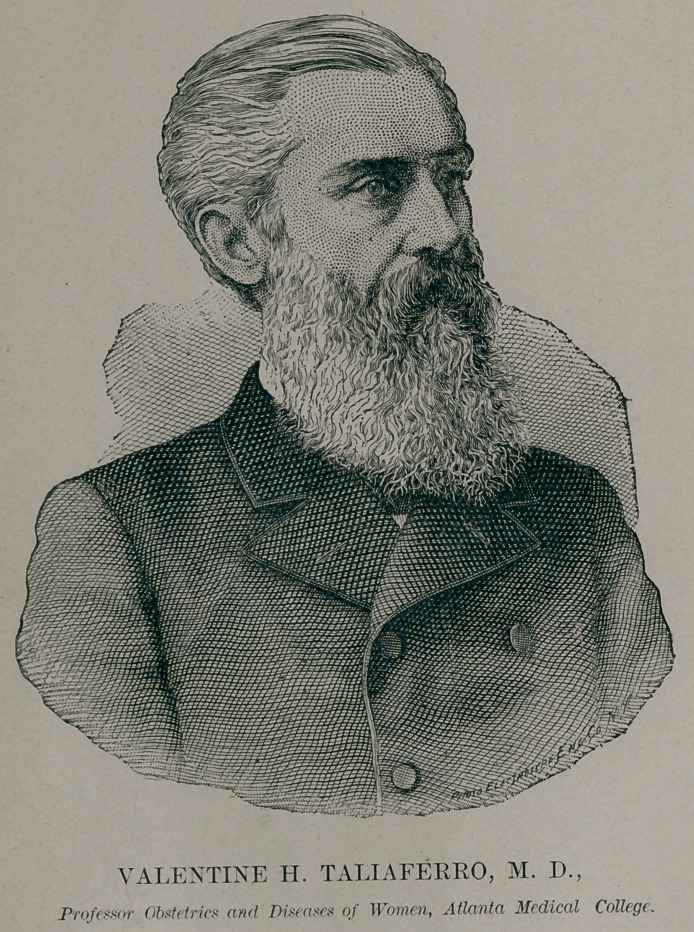# Our Portrait Gallery

**Published:** 1884-07

**Authors:** 


VALENTINE H. TALIAFERRO, M. D.,

### OUR PORTRAIT GALLERY.

### VALENTINE H. TALIAFERRO, M.D.

This eminent gynæcologist, although born in Oglethorpe county, Ga. (September 24th, 1831), comes of remote Italian ancestry, and in his personal appearance and distinguishing traits of character, he displays some of the finest qualities of that gifted race. These ancestors, in coming to America, first settled in the vicinity of Williamsburg, Va.. where they were counted with the wealthy and respectable families of that thriving section of the "Old Dominion." Mr. Zack Taliaferro, his great-grandfather, who lived in Amherst county, was the father of that gallant and distinguished soldier of the Revolutionary war, Col. Benjamin Taliaferro, who won an enviable reputation in many hard fought battles, commanding a company under Gen. Washington during the severe campaigns of 1777–8, in the Jerseys. At the battle of Princeton he forced the surrender of a company of British troops, and ragged and shoeless (as were the American soldiers in that hard struggle), he stepped forward and proudly accepted the surrendered sword of the elegantly uniformed British commander. Later on Col. Taliaferro joined the Southern army and was captured at the siege of Charleston. Returning to Virginia on parol, he resumed the peaceful occupation he had left to serve his country on the battle field.

About the year 1785 Col. Taliaferro removed to Georgia, where his brilliant military reputation and sterling character soon made him one of the foremost men of his day in the State. Among other honors conferred upon him, he was elected a member of Congress, President of the Georgia Senate, a Judge of the Superior Court (although not a lawyer), and Trustee of the State University, in all of which positions he won added reputation as a patriot and statesman. Taliaferro county, the home of the lamented Alexander Hamilton Stephens, will perpetuate his memory far into the distant future. Col. Taliaferro's son, Warren, who located on Broad River, in Oglethorpe county, married a sister of Gov. George R. Gilmer, of this State, and their only son, Col. Charles B. Taliaferro (lately deceased at Columbus), was the honored father of the subject of this sketch. After giving his son the best educational advantages that home institutions offered, he sent him to the University of New York, located in that city, from the medical department of which he graduated in 1852.

Coming back to Georgia, Dr. Taliaferro was united in marriage to Miss Mary A., daughter of Dr. B. O. Jones, of Atlanta, with whom he had studied medicine, and after practicing his profession in Palmetto, Atlanta, and Columbus, he finally returned to Atlanta, where he has since remained. His advancement in honors and reputation has been steady and marked. In 1857 he was elected Vice-President of the Medical Association of Georgia; in 1877 he became President of the Atlanta Academy of Medicine; in 1876 he was a member of the International Medical Congress, held at Philadelphia; also, in 1859 he was elected Professor of Materia Medica, in the Oglethorpe Medical College at Savannah, which he resigned the following year; in 1872 he was chosen Professor of "Diseases of Women and Children," in the Atlanta Medical College, and in 1875 was transferred to the Chair of "Obstetrics and Diseases of Women," which position he now fills with distinguished ability; in 1876 he was Dean of the Faculty, and a year later was made a Trustee. In addition to this he was the efficient Secretary and executive officer of the Georgia State Board of Health, (created in 1875) during its active existence. Being a man of untiring energy and boundless resources, he has been able to respond to the various calls made upon him for such services.

Dr. Taliaferro has made many valuable contributions to medical literature, among the more noted we mention "Medication by the use of Uterine Cloth Tents in Diseases of the Body and Cavity of the Uterus;" "Pathological Sympathies of the Uterus;" "The Corset in its Relation to Uterine Diseases;" "New Intra-Uterine Pessaries:" "The application of Pressure in the Treatment of Disease of the Uterus, Ovaries and Peri-uterine Structures;" "New Vaginal and Intra-uterine Pessaries" etc.

In the spring of 1881 Dr. Taliaferro established his private infirmary for the treatment of diseases of women. This infirmary has grown steadily until now it includes three large two story buildings fitted up in the most approved style.

When the late war commenced Dr. Taliaferro was residing in Columbus, and was one of the first to respond to the call for volunteers. As Surgeon of the City Light Guard of Columbus, he was brought into the Second Georgia Battalion, commanded by Col. Thomas Hardeman, of Macon, of which he was elected Surgeon. Owing to his inheriting a liberal share of the military spirit of his ancestors, Surgeon Taliaferro soon yearned for a more active position, and resigning from the battalion he became Colonel of the Tenth Georgia Cavalry, which he commanded with rare courage and skill, leaving the army at the close of the war with the Brevet rank of Brigadier-General. It was this active service that gave Dr. Taliaferro his present fine military bearing and trained him to quick movements and prompt results. There were few handsomer or more chivalrous soldiers in the Confederate army, and since the war he has held in kind remembrance the gallant men of that bloody period. His "Memorial Address" at the graves of the dead of the City Light Guard, delivered a few years ago at Columbus, was an eloquent and beautiful tribute to the "Lost Cause" and its "Fallen Braves."

In his domestic relations Dr. Taliaferro has been most happily blessed. A devoted wife and four bright, handsome children have made his home life attractive and joyous. His eldest son, Ben. Taliaferro (named after Col. Ben. Taliaferro), although educated at the University of the South, at Sewanee, Tenn., went to Florida and made a delightful winter home for his invalid mother. Rolling up his sleeves and disdaining ease or professional honors, this delicate young man has reared his orange groves, made a success of dairy farming and stock breeding, and proved that fine Jersey cattle can be made profitable in Orange county, Florida. His noble example is worthy of all praise, and should have a good effect upon the young men of the South. The eldest daughter, Mary Taliaferro, has just been united in marriage to Dr. George H. Noble, a brilliant young graduate of the Atlanta Medical College, and at present a partner with Dr. Taliaferro. Few young physicians have started in life with as bright prospects as surround Dr. Noble, and in him Dr. Taliaferro finds an able and trusted assistant, even in his most intricate cases.

## Figures and Tables

**Figure f1:**